# Effect of the Addition of MgO Nanoparticles on the Thermally-Activated Shape Memory Behavior of Plasticized PLA Electrospun Fibers

**DOI:** 10.3390/polym14132657

**Published:** 2022-06-29

**Authors:** Adrián Leonés, Laura Peponi, Stefano Fiori, Marcela Lieblich

**Affiliations:** 1 Instituto de Ciencia y Tecnología de Polímeros (ICTP-CSIC), C/Juan de la Cierva 3, 28006 Madrid, Spain; aleones@ictp.csic.es; 2 Interdisciplinary Platform for “Sustainable Plastics towards a Circular Economy” (SUSPLAST-CSIC), 28006 Madrid, Spain; 3 Condensia Química SA, R&D Department, C/La Cierva 8, 08184 Barcelona, Spain; s.fiori@condensia.com; 4 Centro Nacional de Investigaciones Metalúrgicas (CENIM-CSIC), 28040 Madrid, Spain; marcela@cenim.csic.es

**Keywords:** smart materials, polymer fibers, nanoparticles, electrospinning, shape memory behavior

## Abstract

In this work, the thermally-activated shape memory behavior of poly(lactic acid)-based electrospun fibers (PLA-based efibers) reinforced with different amounts of magnesium oxide (MgO) nanoparticles (NPs) was studied at different temperatures. In particular, MgO NPs were added at different concentrations, such as 0.1, 0.5, 1 and 3 wt%, with respect to the PLA matrix. The glass-transition temperature of PLA-based efibers was modulated by adding a 20 wt% of oligomer lactic acid as plasticizer. Once the plasticized PLA-based efibers were obtained and basically characterized in term of morphology as well as thermal and mechanical properties, thermo-mechanical cycles were carried out at 60 °C and 45 °C in order to study their thermally-activated shape memory response, demonstrating that their crystalline nature strongly affects their shape memory behavior. Importantly, we found that the plastificant effect in the mechanical response of the reinforced plasticized PLA efibers is balanced with the reinforcing effect of the MgO NPs, obtaining the same mechanical response of neat PLA fibers. Finally, both the strain recovery and strain fixity ratios of each of the plasticized PLA-based efibers were calculated, obtaining excellent thermally-activated shape memory response at 45 °C, demonstrating that 1 wt% MgO nanoparticles was the best concentration for the plasticized system.

## 1. Introduction

Among smart materials, shape memory polymers (SMPs) are a very interesting class of self-evolving materials able to recover their original shape from a temporary shape when exposed to external stimuli such as temperature [1], pH [2], light [3] or humidity [4]. In particular, thermally-activated shape memory response is achieved when heating the material up to a transition temperature (T*_trans_*), which is responsible for shape changing [5]. In general, both glass transition temperature (T_g_) and melting temperature of the polymer (T_m_) can be taken as T*_trans_* depending of the nature of the polymer itself. Usually, thermo-mechanical cycles are used in order to study the shape memory response of polymeric materials. In fact, above the T*_trans_*, SMPs can be deformed to a temporary shape under applied external stimulus. Then, after cooling down below the T*_trans_*, the temporary shape is fixed, until finally, heating up again above T*_trans_* allows the polymer to recover its original shape [5]. Extensive research around SMPs has been published in the past few years regarding engineering [3] or biomedical applications [6]. In particular, for biomedical applications, the thermally-activated shape memory behavior has to be achieved at a T*_trans_* closer to human body temperature in order to be used as devices for minimally invasive surgeries [5,7]. In the last decade, SMPs have been processed in different forms, such as films [8] or fibers [5]. In this regard, one of the processing methods to obtain polymeric fibers is electrospinning, where electrospun polymer fibers are obtained from the formation of the Taylor cone of the polymer solution when exposed to an electric field [9,10]. This low-cost and versatile technique allows for the creation of electrospun fibers (efibers) as woven non-woven mats for potential use in a wide variety of fields [11,12,13].

Among polymers used in electrospinning, poly(lactic acid) (PLA) remains attractive for its excellent biocompatibility, biodegradability and low immunogenicity [14,15]. In addition, PLA is completely degradable under physiological conditions into non-toxic products [14,16]. Furthermore, PLA shows thermally-activated shape memory behavior activated for its T_g_, 60 °C in different forms such as films [17] or efibers [5]. On the other hand, PLA shows some mechanical disadvantages. In particular, PLA is brittle and shows poor mechanical performance in terms of ductility and toughness, therefore its direct application for recreating human tissues is limited [11]. Nevertheless, the T_g_ of PLA has to be modulated to lower values when considering its use in biomedical fields, and for this purpose, the use of plasticizer for PLA matrix can be one of the main strategies used [18]. To overcome these disadvantages, different strategies are carried out when processing PLA-based efibers [19]. One of them is the use of a plasticizer, such as acetyl tri-n-butyl citrate, ATBC [20] or poly(ethylene glycol),(PEG) [21] for decreasing the T_g_ in PLA-based efibers. Recently, special attention has been given towards the use of oligomer lactic acid (OLA) as a plasticizer for PLA due to its good compatibility and miscibility [22,23]. Additionally, PLA and OLA degrade in monomeric lactic acid inside the human body, which might not limit the use of OLA as a plasticizer in biomedical fields [24]. Moreover, the use of OLA in thermal-activated shape memory PLA-based efibers has been previously studied by Leones et al. at temperatures close to that of the human body, such as 40 °C and 45 °C, with excellent values of strain fixity and strain recovery ratios [5]. Therefore, the use of OLA as a plasticizer to tailor the T_g_ of PLA can be considered as a successful strategy to obtain electrospun PLA-based fibers.

On the other hand, in order to enhance the mechanical properties of PLA-based efibers, the addition of both organic and inorganic nanoparticles (NPs) has been commonly studied [25,26,27]. Among the inorganic ones, magnesium-based nanoparticles, (Mg-based NPs) are attracting attention for biomedical applications due to the role of magnesium in cellular activities such as the stimulation of bone cell differentiation [11,28], its good mechanical properties [29] and its antimicrobial activity [11].

In this work, a deep study on the thermally-activated shape memory capability of plasticized PLA-based efibers reinforced with MgO NPs at temperatures close to that of the human body has been conducted. Based on our previous work, the amount of OLA was set at 20% in order to properly modulate the T_g_ of PLA and MgO NPs were added at different concentrations, such as 0.1, 0.5, 1 and 3 wt%, with respect to the PLA matrix. Once the PLA-based efibers were obtained, and their diameters, as well as their thermal and mechanical behaviors, were characterized, thermo-mechanical cycles were carried out at 60 °C and 45 °C in order to study their thermally-activated shape memory response and to examine how the addition of both plasticized and nanoparticles can affect its shape memory effect.

## 2. Materials and Methods

Polylactic acid (PLA3051D), 3% of D-lactic acid monomer, molecular weight 14.2 × 10^4^ g·mol^−1^, density 1.24 g·cm^−3^) was supplied by NatureWorks^®^. Lactic acid oligomer (Glyplast OLA8, ester content > 99%, density 1.11 g·cm^−3^, viscosity 22.5 mPa·s, molecular weight 1100 g·mol^−1^) was kindly supplied by Condensia Quimica SA. Chloroform, CHCl_3_, (99.6% purity) and *N,N*-dimethylformamide, DMF, (99.5% purity) from Sigma Aldrich were used as solvents. Magnesium oxide nanoparticles (MgO NPs, average particle size of 20 nm, 99.9% purity, molecular weight 40.30 g∙mol^−1^) were supplied by Nanoshel LLC, Wilmington, DE, USA.

Previous to the electrospinning process, each solution was prepared according to the following steps. Firstly, the corresponding amounts of PLA and OLA were dissolved separately in CHCl_3_ and stirred overnight at room temperature. Secondly, the amount of MgO NPs was weighed and dispersed in 20 mL of CHCl_3,_ then, after 30 min, the OLA solution was added and dispersed for 60 min. Afterwards, the PLA solution was added and dispersed for another 60 min. Finally, we added the necessary volume of DMF to assure the proportion of solvents CHCl_3_:DMF (4:1). The dispersion process was carried out with a sonicator tip (Sonic Vibra-Cell VCX 750, Sonics & Materials, Newton, CT, USA) of 750 watts and an amplitude of 20%. Once the different solutions were obtained, electrospun fiber mats were prepared in an Electrospinner Y-flow 2.2.D-XXX (Nanotechnology Solutions) following our previously described method [30]

Scanning Electron Microscopy (SEM) (PHILIPS XL30 Scanning Electron Microscope) was used in order to study the morphology and the diameters of the efibers. All the samples were previously gold-coated (~5 nm thickness) in a Polaron SC7640 Auto/Manual Sputter. Diameters were calculated as the average value of 30 random measurements for each sample using ImageJ software. The distribution of MgO NPs into the electrospun fibers was studied by field emission scanning electron microscopy, FESEM, (Hitachi S8000).

Thermal transitions were studied by Differential Scanning Calorimetry, DSC, in a DSC Q2000 TA Instrument under nitrogen atmosphere (50 mL∙min^−1^). The thermal analysis was programmed at 10 °C∙min^−1^ from −60 °C up to 180 °C obtaining the glass transition temperature (T_g_) calculated as the midpoint of the transition, the melting temperature (T_m_), the cold crystallization enthalpy (ΔH_cc_) and the melting enthalpy (ΔH_m_). The degree of crystallinity (X_c_%) was calculated using the equation 1, taking the value of crystallization enthalpy of pure crystalline PLA (ΔH_m_°) as 93.6 J∙g^−1^ and W_f_ as the weight fraction of PLA in the sample [31].

(1) $$X_{c}\left(\%\right)=\frac{{\Delta H}_{m}-{\Delta H}_{\mathrm{cc}}}{{\Delta H}_{m}{}^{\circ}}\times \frac{1}{W_{f}}\times 100$$

Mechanical properties were evaluated by tensile test in a QTest™ 1/L Elite instrument equipped with a 100 N load cell at room temperature. Strain rate and initial length between clamps were set at 10 mm∙min^−1^ and 10 mm, respectively. Five samples of 20 mm length, 6 mm width and 100 µm of average thickness were measured. The toughness was measured by the area under the stress–strain curve for each sample. The mechanical properties were statistically analyzed by one-way analysis of variance (ANOVA) and Tukey’s test with a 95% confidence level, using the statistical computer package Statgraphics Centurion XVII (Statpoint Technologies, Inc., Warrenton, VA, USA) [32].

Dynamic Mechanical Thermal Analysis (DMTA) was carried out in a DMA Q800 TA instrument in order to study their thermally-activated shape memory properties. At least four different thermo-mechanical cycles were performed for each sample. Programming as well as recovering stages were set by three different steps: (1) Isotherm at Ttrans during 5 min (T*_trans_* = 60 °C and 45 °C) and then, a ramp stress of 0.2 MPa∙min^−1^ was applied until ε = 50%. (2) Cool down at 10 °C under constant stress. (3) After releasing the stress at 0.50 MPa∙min^−1^, the sample was heated at 3 °C∙min^−1^ up to T*_trans_* and maintained for 30 min. Strain fixity ratio (R_f_) and the strain recovery ratio (R_r_) were calculated by Equations (2) and (3):

(2) $$R_{f}\left(N\right)=100\times \frac{\varepsilon_{u}\left(N\right)}{\varepsilon_{m}\left(N\right)}$$

(3) $$R_{r}\left(N\right)=100\times \frac{\varepsilon_{m}\left(N\right)-\varepsilon_{p}\left(N\right)}{\varepsilon_{m}\left(N\right)-\varepsilon_{p}\left(N-1\right)}$$

where ε_m_ is the maximum strain after cooling to T_fix_ and before releasing the stress, ε_u_ is the fixed strain after releasing the stress at T_fix_ and ε_p_ is the residual strain after retaining the sample at T*_trans_* for 30 min [3].

## 3. Results and Discussion

**Figure 1:**
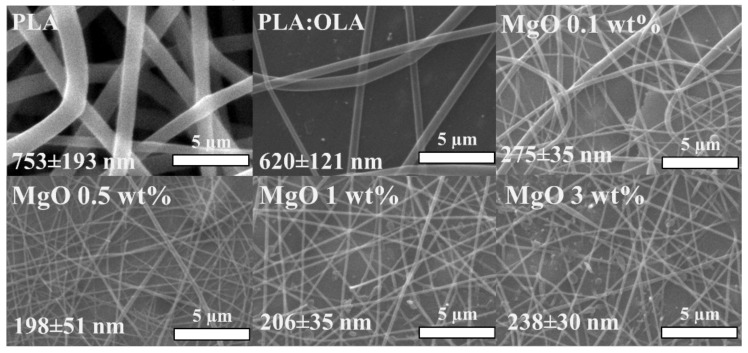
SEM images of PLA, PLA:OLA and MgO 0.1, 0.5, 1 and 3 wt%.

After obtaining the PLA-based polymer solutions, the corresponding woven non-woven efibers mats were properly obtained by electrospinning, and their morphology was studied by scanning electron microscopy (SEM). In Figure 1, SEM images for neat PLA, PLA:OLA and 0.1, 0.5, 1 and 3 wt% MgO PLA-based efibers are shown with their corresponding average diameter values. As can be seen, straight and randomly-oriented efibers were properly obtained. From the diameter point of view, the addition of OLA decreases the average diameter from 753 ± 193 nm for neat PLA to 620 ± 121 nm for PLA:OLA efibers. As expected, the addition of 20% of OLA provokes a reduction in the average diameter of about 18% which is in accordance with our previous work [5]. The average diameter values for the efibers with different amounts of MgO NPs were measured and reported in Figure 1. When adding 0.1 wt% MgO NPs the reduction of diameter dimension is quite good, obtaining an average value of 275 ± 35 nm, a reduction of about 55% with respect to the diameter obtained for PLA:OLA efibers. When 0.5 and 1 wt% MgO NPs have been added, the reduction of the average diameters increased obtaining values of 198 ± 51 and 206 ± 35 nm, respectively, corresponding to a reduction of about 70% with respect to PLA:OLA efibers. Finally, increasing the amount of NPs to 3 wt%, their average diameter slowly increased to 238 ± 30 nm, indicating a reduction of more than 60% with respect to the diameter value of PLA:OLA efibers. In general, as previously reported in literature, the addition of NPs tends to decrease the average diameter of fibers [33]. Leonés et al. studied the evolution of the diameter with both, organic and inorganic NPs dispersed in PLA at 1 wt%, and reported a reduction of 60% with cellulose nanocrystals, 40% with chitosan and 10% with Ag NPs [32].

**Figure 2:**
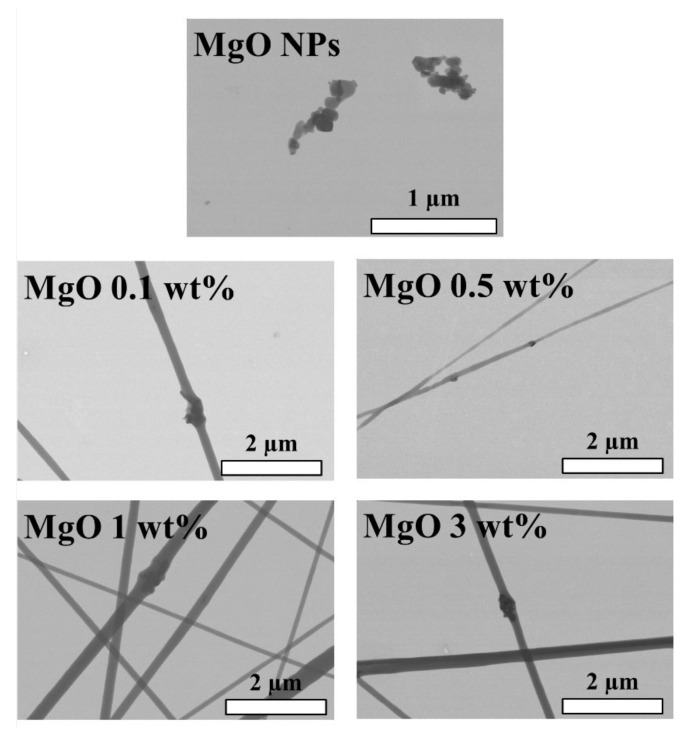
FESEM images of MgO NPs as well as MgO 0.1, 0.5, 1 and 3 wt% efibers.

Additionally, the distribution of MgO NPs into the PLA-based efibers was studied by FESEM. In Figure 2, FESEM images of each of the PLA-based efibers, as well as MgO NPs, are shown. First of all, it is important to note the proper incorporation of MgO NPs into the PLA fibers during the electrospinning process. In fact, MgO NPs can be observed in each PLA-based efiber formulation confirming that they are properly embedded into the electrospun polymeric fibers. It is worth noting that no agglomerations of MgO NPs are observed, considering the average diameter of efibers and their distributions throughout the efibers.

**Figure 3:**
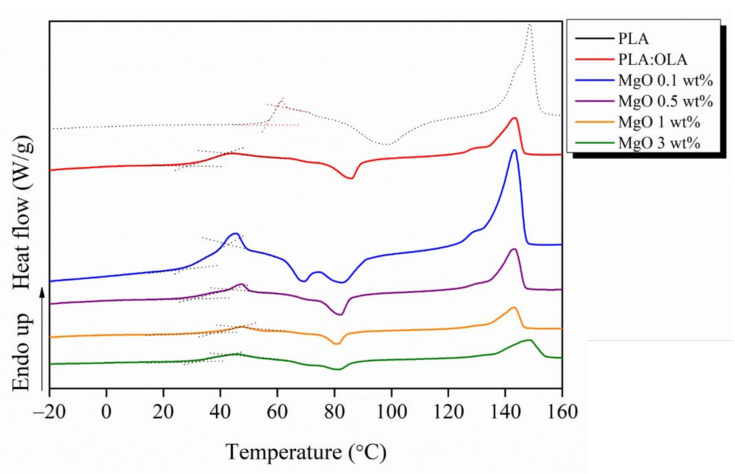
DSC thermograms for PLA-based efibers.

Once the morphology of the fibers was characterized, their thermal characterization was carried out. DSC thermograms for PLA-based efibers are shown in Figure 3. As expected, the addition of 20% of OLA clearly decreased the T_g_ of the efibers from 60 °C for neat PLA to 36 °C for PLA:OLA sample. OLA properly decreases the T_g_ of PLA to values closer to the physiological temperature. However, the addition of MgO NPs in the range of 0.1–3 wt% does not significantly affect the T_g_ of the reinforced efibers [30].

As previously reported [22], the short polymeric chains tend to crystallize, thus enhancing the formation of PLA crystals, which leads to slightly higher degrees of crystallinity (X_c_). However, PLA:OLA efibers show a degree of crystallinity of 1.1% which indicates an almost amorphous material as well as the neat PLA efibers. Furthermore, as reported in the scientific literature, the addition of NPs can affect the crystallization behavior of polymeric matrices due to their nucleation effect [34,35]. In our case, the addition of MgO NPs strongly affects the crystallinity of the system. In particular, for concentrations 0.1 and 0.5 wt%, two cold crystallization peaks were observed at 66 and 81 °C. Once this amount of NPs is overcome, only a single cold crystallization peak was observed for MgO 1 and 3 wt% at 82 °C. Therefore, the nucleation effect of MgO NPs is clear having strongly increased the degrees of crystallinity to 27.6, 27.3, 25.4 and 23.2% for MgO 0.1, 0.5, 1 and 3 wt%, respectively. It is important to remark how the lowest amount of MgO yields to the highest X_c_ of 27.6%. This fact will strongly affect the thermally-activated shape memory response of the reinforced efibers, as discussed later.

**Figure 4:**
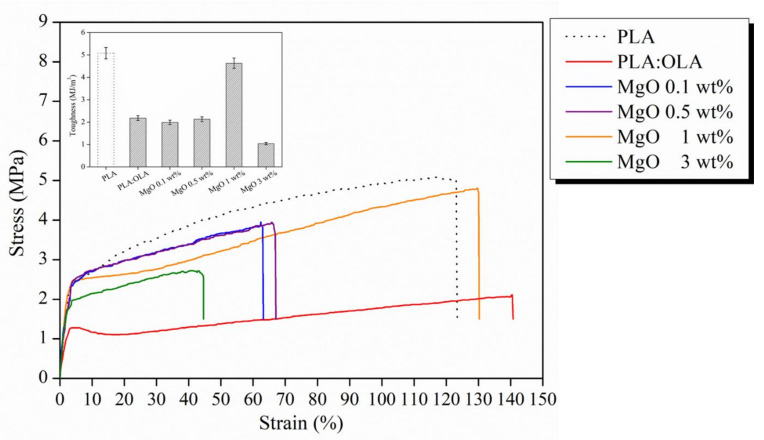
Stress–strain curves for PLA-based efibers. Inset are the toughness values.

From the mechanical point of view, elastic modulus (E), tensile strength (σ) and elongation at break (ε) were calculated from the tensile stress–strain test and summarized in Table 1 for PLA:OLA and the reinforced PLA-based efibers. Additionally, the stress–strain curves for each PLA-based efibers mats are showed in Figure 4.

As reference, the values obtained for neat PLA efibers are also indicated in brackets. However, in our case, PLA:OLA has to be considered as the neat matrix for the reinforced electrospun fibers. Statistical ANOVA analysis was carried out between samples in order to set statistical differences in the mechanical performance of PLA-based efibers. As previously reported, the addition of OLA clearly decreases the mechanical response of PLA efibers in terms of elastic modulus as well as of tensile strength [5]. It is possible to note how the addition of MgO NPs, at every concentration used, strongly increases the mechanical response of our plasticized system. In particular, the addition of MgO significantly increases the E of efibers in the entire range of 0.1–3 wt% in comparison with PLA:OLA efibers yielding values comparable with unplasticized PLA efibers. In fact, as can be seen in Table 1, an increase in the E values of more than 40% for all the different concentrations of MgO, with respect to PLA:OLA was observed and confirmed by ANOVA analysis. Therefore, we can conclude that the plastificant effect of OLA is balanced by the reinforced effect of the addition of NPs to the PLA-based efibers.

Not only is the elastic modulus positively affected by the addition of MgO, the tensile strength of the electrospun fibers is positively affected as well. In fact, the tensile strength values increase up to 4.2 ± 0.8 MPa when 1 wt% of MgO is added, which means an increase of 68% with respect to PLA:OLA efibers, and also slightly increases with respect to neat PLA efibers. For the elongation at break, only MgO at 1 wt% shows a very similar behavior with respect to the neat PLA. For the other studied concentrations, more brittle systems with very low values of elongation at break have been obtained. This fact is strongly correlated with the crystallinity of the system. In fact, in the samples with higher degrees of crystallinity, that is MgO 0.1 and 0.5 wt%, the elongation at break falls down to 62 ± 3 and 64 ± 5%, respectively. That means it falls to half the value of the PLA:OLA matrix. However, our best formulation is obtained for PLA-based efibers reinforced with 1 wt% MgO, showing the best mechanical performance. Thus, the addition of MgO 1 wt% not only improved the elongation at break up to 130 ± 30% but also its toughness, changing value from 2.18 ± 0.11 MJ/m^3^ for our polymeric matrix PLA:OLA to 4.63 ± 0.23 MJ/m^3^ for MgO 1 wt%, which is in the same range of toughness values for neat PLA efibers, 5.08 ± 0.25 MJ/m^3^, as can be seen in the inset of Figure 4.

Furthermore, it is worth noting that the mechanical properties of our efibers are comparable with those of human tissues, such as the aortic valve (E = 2–15 MPa), mitral valve anterior leaflet (E = 3.6 ± 1.8 MPa) and human skin (E = 3–54 MPa, σ = 1–20 MPa, ε at break = 30–70%), which indicates they could present a potential use in medical fields [11].

Once the thermal and mechanical properties of the efibers were considered, their thermally-activated shape memory behavior was studied at 50% of deformation and 60 °C and 45 °C as transition temperatures. We chose these temperatures considering that we know PLA is able to show thermally-activated shape memory response at 60 °C, even if it is obtained by electrospinning [5]. However, we previously reported that by adding 20 wt% of OLA, we were able to decrease the shape memory response temperature of the efibers down to 45 °C, thus we consider their T_g_ as the temperature able to activate their shape memory behavior. As previously reported [5], for a T*_trans_* = 60 °C, PLA efibers properly fix the temporary shape with a R_f_ between 96 and 99%, as well as recover its original shape at 60 °C with R_r_ higher than 88%, showing the excellent capability of PLA to present thermally-activated shape memory behavior at 60 °C, even in the form of electrospun fibers [5]. The values for the strain fixity ratio (R_f_) and the strain recovery ratio (R_r_) for the reinforced systems are reported in Table 2.

**Figure 5:**
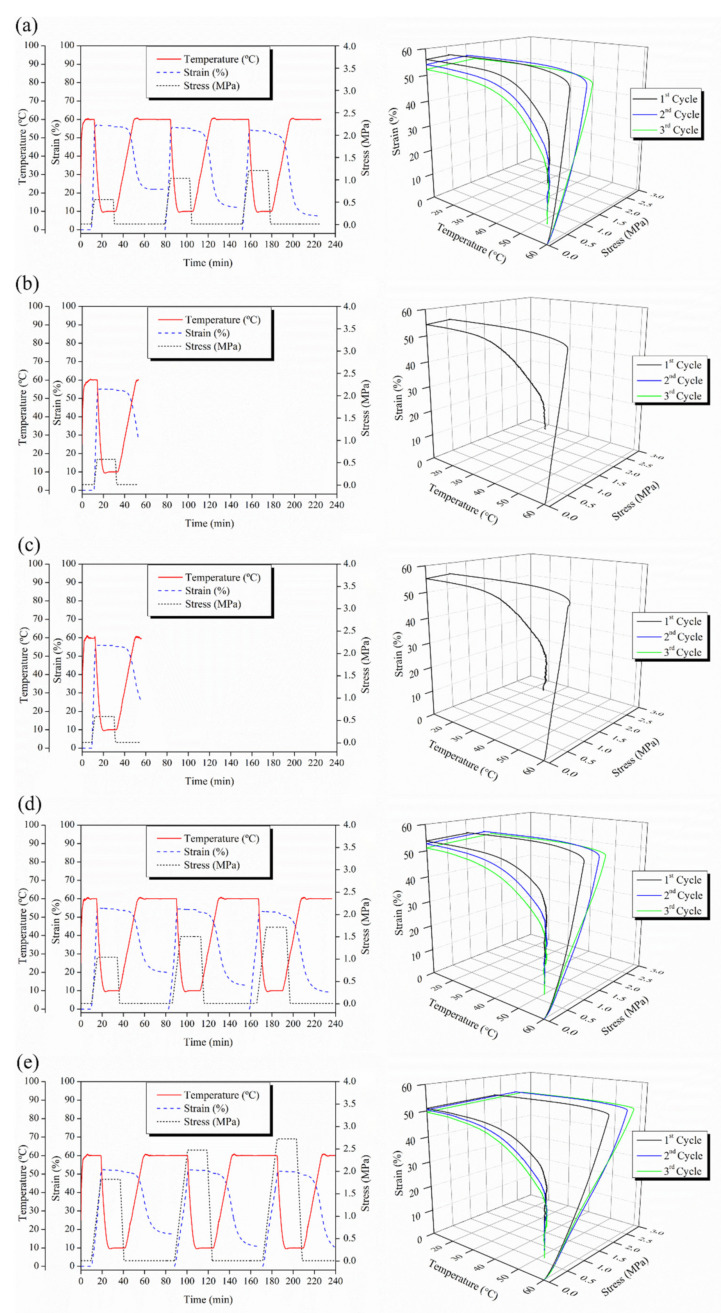
2D and 3D thermo-mechanical cycles performed at 60 °C for (**a**) PLA:OLA, (**b**) MgO 0.1 wt%, (**c**) MgO 0.5 wt%, (**d**) MgO 1 wt% and (**e**) 3 wt% MgO efibers.

In Figure 5, 2D and 3D thermo-mechanical cycles performed at 60 °C for PLA:OLA (Figure 5a) and for the reinforced PLA-based efibers (Figure 5b,e) are reported.

PLA:OLA efibers are able to show good shape memory response at 60 °C, as indicated in Figure 5a, even if the R_r_ is not very high. As previously indicated, the activation temperature of 60 °C is too high for this system [5].

PLA-based efibers reinforced with low amount of MgO NPs, that is 0.1 and 0.5 wt%, do not show thermally-activated shape memory behavior, while PLA-based efibers reinforced with 1 and 3 wt% of MgO nanoparticles show thermally-activated shape memory response at 60 °C. In particular, R_f_ values higher than 97% were obtained for both MgO 1 and 3 wt%, showing an excellent capability to fix the temporary shape. For MgO 1 and 3 wt%, however, R_r_ values of 64 and 66% were obtained after the first cycle, which evidences a poor capability of recovering the original shape. In fact, taking into account that the plasticized matrix PLA:OLA presents T_g_ of 36 °C, the temperature needed for the activation of the shape memory behavior is lower than 60 °C. As can be observed in Figure 5d,e, the recovering stage starts close to 40 °C. This temperature is in agreement with the T_g_ values of efibers measured by DSC.

**Figure 6:**
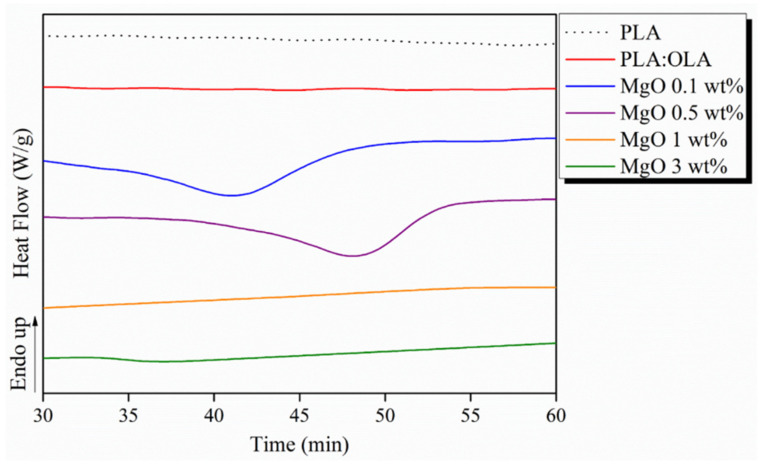
DSC thermograms for PLA-based efibers.

As said before, it is worth noting that MgO 0.1 and 0.5 wt% efibers do not show thermally-activated shape memory behavior at 60 °C, as observed in Figure 5b,c, respectively. It is important to remark that in these cases, T*_trans_* = 60 °C is very close to the cold crystallization peak observed by DSC for MgO 0.1 and 0.5 wt%, and their crystallizations do not allow the shape memory response of these samples. In order to verify this point, isothermal crystallization at 60 °C for PLA-based efibers after 60 min was studied by DSC and reported in Figure 6.

As can be observed, a broad crystallization peak appears for MgO 0.1 and 0.5 wt% efibers, respectively, at 60 °C, avoiding their shape memory response at this temperature. This nucleating effect at a low amount of NPs in the PLA matrix has been widely described in scientific literature. For instance, *Tarani* et al. studied nanocomposites of PLA with zinc oxide, titanium dioxide and silver NPs, and reported that the NPs acted as heterogeneous nucleating agents, accelerating the cold crystallization of PLA [36].

**Figure 7:**
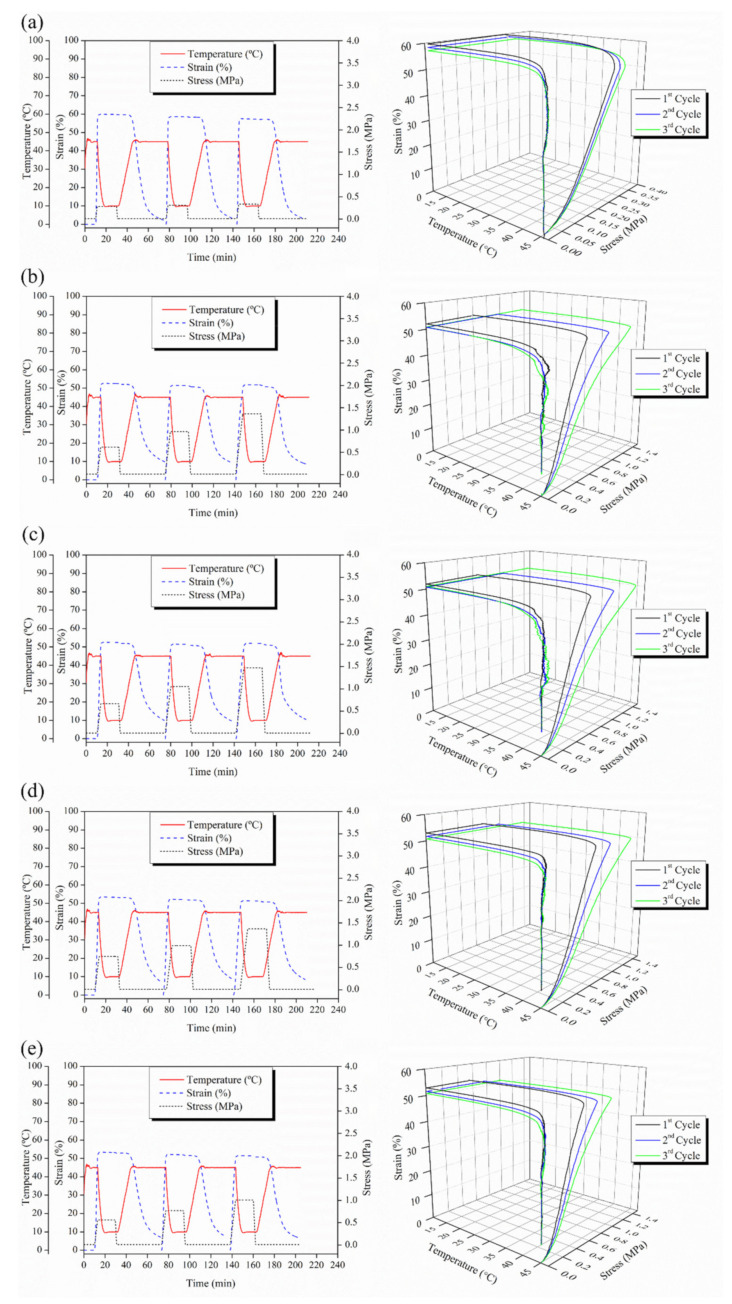
2D and 3D thermo-mechanical cycles performed at 45 °C for (**a**) PLA:OLA, (**b**) MgO 0.1 wt%,(**c**) MgO 0.5 wt%, (**d**) MgO 1 wt% and (**e**) MgO 3 wt% efibers.

Taking into account their potential applications in biomedical fields, the thermally-activated shape memory behavior was studied at 45 °C and their thermal-mechanical cycles are reported in Figure 7.

As expected, neat PLA efibers do not show thermally-activated shape memory at 45 °C, while, as described in our previous work, PLA:OLA efibers showed excellent shape memory behavior at 45 °C with R_f_ higher than 95% and R_r_ of 100% after each thermo-mechanical cycle [5]. At this temperature, all the reinforced efibers also showed excellent capability to fix the temporary shape at 45 °C with Rf higher than 99% for the first thermo-mechanical cycle maintaining quite constant values in the other cycles. Additionally, R_r_ values higher than 82% were achieved for each sample during all the shape memory thermo-mechanical cycles. Thus, the addition of MgO NPs in the range of 0.1–3 wt% does not cause the loss of the thermally-activated shape memory behavior of PLA:OLA matrix at 45 °C, showing excellent R_r_ and R_f_ values comparable with those of PLA:OLA efibers.

**Figure 8:**
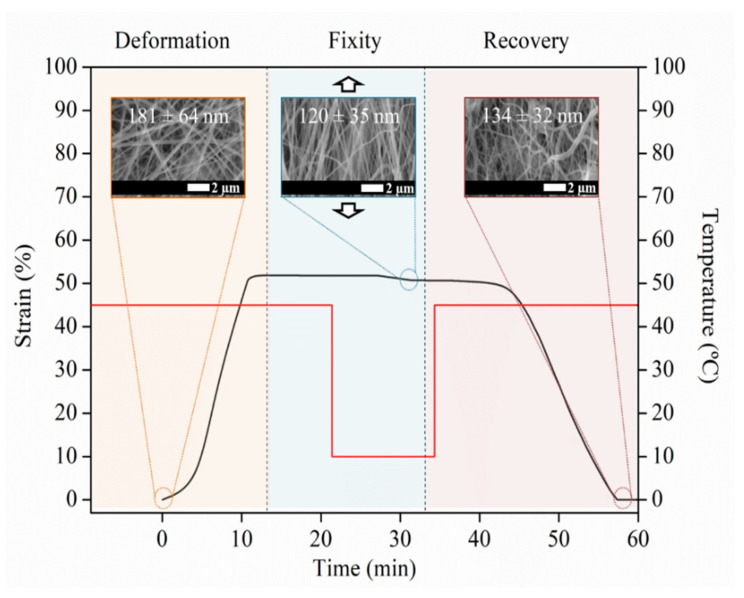
Schematic representation of the first thermo-mechanical cycle for MgO 1 wt% efibers and SEM images at different stages.

In order to study how the thermo-mechanical cycles can affect the morphology of the randomly oriented efibers mats, SEM analysis was carried out in the MgO 1 wt% specimen at the different stages of the shape memory test at 45 °C and reported in Figure 8. Initially, randomly oriented efibers can be observed with average diameter of 181 ± 64 nm. When the programming step is finished, the temporary shape is fixed, and, as expected, the efibers are clearly oriented in the direction of the force applied during the programming step, decreasing the average diameters of efibers to 120 ± 35 nm, which supposes a reduction of 34% with respect to the initial average diameter.

In the recuperation step, the efibers are heated again up to 45 °C, showing an average diameter of 134 ± 32 nm, which supposes a slight increase with respect to the values obtained in the fix-shape stage, but the orientation is maintained.

**Figure 9:**
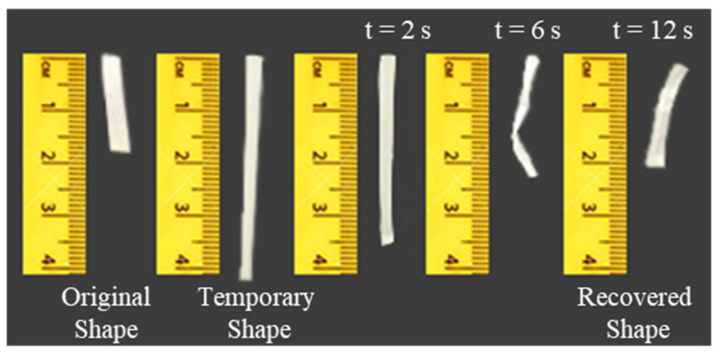
Macroscopic demonstration of the thermally-activated shape memory response at 45 °C for PLA-based efibers reinforced with 1 wt% MgO NPs.

Finally, the thermally-activated shape memory behavior of MgO 1 wt% efibers was proven macroscopically as shown in Figure 9. In particular, a specimen of 20 mm length, 4 mm width and 100 µm of average thickness was programmed by deforming the sample at 45 °C up to 40 mm length, that is a deformation of 100%, and cooling down in order to fix the temporary shape. Once programmed, the sample was reheated at 45 °C. Pictures were taken at different recovery times in order to visualize its effect. As shown in Figure 9, for MgO 1 wt% efibers, the recovery starts after 2 s, taking only 12 s to totally recover the original shape at 45 °C.

## 4. Conclusions

In this work, plasticized PLA-based efibers reinforced with different amounts of MgO nanoparticles have been successfully obtained. The mechanical response of the plasticized reinforced system is compared with unplasticized PLA efibers but with a T_g_ lower than 20 °C, obtaining PLA-based efibers reinforced with 1 wt% MgO NPs as the best formulation, with a toughness comparable to that of neat PLA efibers. Moreoverfrom the mechanical point of view, the plasticizer effect due to the addition of plasticizer is strongly balanced by the reinforced effect due to the addition of nanoparticles. Furthermore, all the reinforced systems are able to show excellent thermally-activated shape memory response at 45 °C. Therefore, in this study, we have modulated the T_g_ of PLA systems to values close to the human body without loss of their mechanical response and without loss of their thermally-activated shape memory behavior.

## Figures and Tables

**Table 1:** Mechanical properties for each PLA-based efibers mats. Different letters in the column indicate significant differences according to Tukey’s test (*p* < 0.05). * Values are significant at *p* < 0.05.

Sample	E (MPa)	σ (MPa)	ε at Break (%)
PLA:OLA (PLA)	64.0 ± 6.0 ^b^ (91.0 ± 8.0 ^a^)	2.5 ± 1.0 ^c^ (3.8 ± 0.5 ^a^)	140 ± 27 ^a^ (135 ± 10 ^a^)
MgO 0.1 wt%	91.4 ± 7.9^a^	3.6 ± 0.4 ^b^	62 ± 3 ^b^
MgO 0.5 wt%	93.3 ± 7.7 ^a^	3.6 ± 0.3 ^b^	64 ± 5 ^b^
MgO 1 wt%	94.4 ± 16.8 ^a^	4.2 ± 0.8 ^a,b^	130 ± 15 ^a^
MgO 3 wt%	90.9 ± 14.6 ^a^	2.7 ± 0.3 ^c^	48 ± 11 ^c^
F ratio	4.09	15.99	61.80
*p*-Value	0.0071 *	0.0000 *	0.0000 *

**Table 2:** Values of strain recovery ratio and strain fixity ratio at different temperatures.

	T*_trans_* = 60 °C						T*_trans_* = 45 °C					
	R_r_ (%)			R_f_ (%)			R_r_ (%)			R_f_ (%)		
Cycles	1st	2nd	3rd	1st	2nd	3rd	1st	2nd	3rd	1st	2nd	3rd
PLA	89	88	88	96	99	99	-	-	-	-	-	-
PLA:OLA	61	71	82	90	93	96	100	100	100	100	97	95
MgO 0.1 wt%	-	-	-	-	-	-	82	83	84	99	99	98
MgO 0.5 wt%	-	-	-	-	-	-	82	83	82	99	99	98
MgO 1 wt%	64	77	83	99	97	97	88	84	84	99	99	99
MgO 3 wt%	66	80	84	98	97	97	87	87	87	99	99	99

## Data Availability

Not applicable.
